# Bionanocomposite Based on Cassava Waste Starch, Locust Bean Galactomannan, and Cassava Waste Cellulose Nanofibers

**DOI:** 10.3390/foods13020202

**Published:** 2024-01-08

**Authors:** Pãmella Fronza, Michelle J. P. A. Batista, Adriana S. Franca, Leandro S. Oliveira

**Affiliations:** 1 Programa de Pós-Graduação em Ciência de Alimentos, Universidade Federal de Minas Gerais, Av. Antônio Carlos 6627, Belo Horizonte 31270-901, MG, Brazil; pamellafronza@hotmail.com (P.F.); michelle.jp.azevedo@gmail.com (M.J.P.A.B.); leandro@demec.ufmg.br (L.S.O.); 2 Departamento de Engenharia Mecânica, Universidade Federal de Minas Gerais, Av. Antônio Carlos 6627, Belo Horizonte 31270-901, MG, Brazil

**Keywords:** bionanocomposites, cassava waste, cellulose nanofibers, polysaccharides, starch, biodegradation

## Abstract

Natural polysaccharides are among the renewable sources with great potential for replacing petroleum-derived chemicals as precursors to produce biodegradable films. This study aimed to prepare biopolymeric films using starch extracted from the periderm and cortex of cassava roots (waste from cassava root processing), locust bean galactomannan, and cellulose nanofibers also obtained from cassava waste. The films were prepared by casting, and their physicochemical, mechanical, and biodegradability properties were evaluated. The content of cellulose nanofibers varied from 0.5 to 2.5%. Although the addition of cellulose nanofibers did not alter the mechanical properties of the films, it significantly enhanced the vapor barrier of the films (0.055 g mm/m^2^ h kPa–2.5% nanofibers) and their respective stabilities in aqueous acidic and alkaline media. All prepared films were biodegradable, with complete degradation occurring within five days. The prepared films were deemed promising alternatives for minimizing environmental impacts caused by the disposal of petroleum-derived materials.

## 1. Introduction

Food products are commercialized in different forms, either in solid, liquid, or semi-solid states, and thus must be packed accordingly to be protected from microbes and other contaminants. Plastics play a significant role in food packaging, and the need to mitigate environmental problems associated with their disposal has prompted research on ecologically safe packaging materials [1]. One of the possibilities to address this situation is using polysaccharides, which are abundant in agri-food products and respective wastes. Several studies have explored using materials such as corn starch, banana peel, chitosan, cassava starch, and galactomannans to find potential ecological alternatives for developing packaging materials [2,3,4,5,6,7]. Many of these materials are blended to primarily improve gas barrier and mechanical properties [8]. These blends are promising because starches and flours result in brittle and highly permeable materials, directly affecting their end-use. Thus, improving the properties of starch and flour-based films is an ongoing challenge. In addition to polysaccharide blends, it is possible to incorporate other components compatible with the polymeric matrix, such as reinforcing agents [9].

Nanofibers extracted from various agri-food wastes have been reported as promising nanofiller reinforcements for packaging materials, including cellulose nanofibers derived from waste streams [10], peach palm residues [4], canola straw [11], and cassava bagasse [12], among others. These studies reported improvements in water vapor permeability and other essential properties of packaging materials, such as moisture and elasticity. For instance, sugarcane bagasse was examined by Ghaderi et al. [13] for the production of nanocomposites. The authors reported that this residue can be converted into high-performance packaging materials. The resulting nanofibers exhibit barrier potential, forming protective films that can be employed in the food industry. However, the extraction of nanofibers still presents a challenge regarding commercial production, especially cost. For this reason, these studies have aimed to simplify the extraction process of these materials by utilizing other lignocellulosic sources, such as agricultural residues [14].

In view of the above, the present study aimed to develop a polymeric film based on a blend of locust bean galactomannans and cassava peel polysaccharides incorporated with cellulose nanofibers extracted from cassava peels. The prepared films were characterized by their mechanical, barrier, chemical, and structural properties.

## 2. Materials and Methods

### 2.1. Materials

Cassava periderm and cortex of different varieties were obtained from a local market. The following reagents were used for nanofiber preparation, potassium hydroxide, acetic acid, EDTA, sodium hydroxide, 50% (*v*/*v*) hydrogen peroxide solution, diethylenetriaminepentaacetic acid (Sigma-Aldrich, St. Louis, MO, USA), magnesium sulfate, and hemicellulase from *Aspergillus niger* (Sigma-Aldrich, St. Louis, MO, USA), with enzymatic activity of 72 U/mL, in a sodium citrate buffer (pH 4.8). The reagents for the production of films were locust bean gum (Sigma-Aldrich, St. Louis, MO, USA) and glycerin P.A. 3-5-dinitrosalicylic acid (Sigma-Aldrich, St. Louis, MO, USA), anhydrous calcium chloride, and hydrochloric acid were used to characterize films and nanofibers. All the reagents used in the present work were purchased from Anidrol (Diadema, Brazil), Neon (Suzano, Brazil), Synth (Diadema, Brazil), and Sigma-Aldrich (São Paulo, Brazil).

### 2.2. Polysaccharide Extraction and Production of Cellulose Nanofibers

Cassava polysaccharides were extracted from the cassava peels following the methodology presented in our previous study [15]. The peels were comminuted in aqueous media in an industrial blender at a ratio of peel to water of 1:1 for 5 min, followed by homogenization of the resulting paste in an agitator for 30 min. The homogenized paste was filtered through a polypropylene mesh, and the filtrate solution was decanted at 7 °C for 12 h. The resulting supernatant was discarded, and the decanted solids were washed several times. Then, the insoluble material (IM) was dried at 50 °C for 24 h, followed by maceration in a 100-mesh sieve (Tyler series). The obtained powdered product was used for film preparation and to produce cellulose nanofibers.

### 2.2.1. Pre-Treatment

The chemical treatment of the powder for nanofiber preparation was carried out according to Andrade-Mahecha et al. [16]. Fifty grams (dry weight) of IM was soaked in 1000 mL of KOH 5% solution. The sample was subjected to mechanical stirring at room temperature for 15 h. The suspension was filtered, and successive washings using deionized water were performed until there was no change in color. The resulting material was diluted with distilled water, and the pH was adjusted to 5 by adding 10% acetic acid. Subsequently, the suspension was submitted to a chelation treatment with EDTA at 70 °C for 1 h to remove metallic ions [17]. The treated material was filtered and washed several times using distilled water until there was no change in color and dried in an oven at 45 °C for 24 h. The insoluble material was added to a hydrogen peroxide solution (%) with three reagents: NaOH (2%), DTPA (0.2%) (diethylenetriaminepentaacetic acid), and MgSO_4_ (3%). The suspension was stirred at 90 °C for 3 h. Again, the material was exhaustively washed using deionized water and filtered until no changes in color could be observed.

#### 2.2.2. Enzymatic Hydrolysis

Preliminary experimental tests were performed to select the best enzymatic hydrolysis conditions. Erlenmeyer flasks containing the substrate (sample from chemical pre-treatment) and sodium citrate buffer (pH 4.8) were placed in an orbital shaker (50 °C, 155 rpm) for 15 min. Subsequently, hemicellulase (*Aspergillus niger*—concentration of 72 U/g of bran) was added to the mixture and stirred for 24 h. The suspensions were placed in a bath at 80 °C for 30 min for enzyme denaturation. Afterward, the remaining powder was rinsed with deionized water. The insoluble material was then separated via filtration and subsequently suspended in ultrapure water. At the end of these procedures, a colloidal suspension of CNFs (cellulose nanofibers) was obtained and stored at 5 °C in a sealed container. Later, the suspensions composed of nanofibers were processed in a high-pressure homogenizer (QR 500 W Ultranique, ECO-SONICSP, Brazil) for five minutes with a power of 500 W to separate the nanofibers from each other and reduce their size. The samples were again stored at 5 °C for further application.

### 2.3. Production of Films

The films were prepared by casting. The control film, BC (biocomposite), was prepared as follows. Cassava peel (periderm + cortex) starch (2.25 g) and locust bean gum (LBG) (0.75 g) were dissolved in 200 mL distilled water under continuous stirring for 45 min at room temperature. Subsequently, 0.6 g of glycerol was added to the suspension, followed by constant stirring for 2 h at 70 °C. For the films with added nanofibers, namely bionanocomposite films, nanofiber suspension was added to the filmogenic solution at different concentrations (0.5, 1.5, and 2.5%). These concentrations were established in preliminary tests, with concentrations higher than 2.5% leading to films with starch agglomerations. The film-forming solutions were placed in an ultrasonic bath (Unique-Ultra Cleaner, model 1650A) for one hour to remove bubbles from the solutions. Subsequently, 150 mL of film-forming solution was transferred to square silicone molds, which were placed in a climate-controlled atmosphere (23 °C and 45% RH) for 24 h, allowing the solvent to evaporate. Following solvent evaporation, the materials were carefully removed from the molds and stored at room temperature. Prior to further analysis, the film samples were preconditioned at 50% relative humidity, at 23 °C, for 48 h.

### 2.4. Characterization of Nanofibers

#### 2.4.1. Yield, Reducing Sugars, and Lignin Contents

Nanofiber yield was calculated from a mass balance, considering the initial mass after alkaline and hydrogen peroxide treatment and the final mass after enzymatic hydrolysis. Analyses of reducing sugars and lignin determination were carried out to characterize the nanofibers. For such, an aliquot of the solution containing nanofibers after enzymatic hydrolysis was used to quantify reducing sugars and thus define the ideal enzyme concentration. After preliminary tests, the following amounts of hemicellulase were selected: 0.046, 0.1, and 0.37 g [18]. The reducing sugar content was determined by the DNS method (3-5-dinitrosalicylic acid), employing a glucose-based calibration curve [19]. The samples were analyzed using a UV-Vis spectrophotometer (Gold brand, model Spectrumlab 53) at a wavelength of 540 nm. Lignin determination was conducted according to the Klason protocol with modifications. 

#### 2.4.2. Atomic Force Microscopy (AFM)

Visualization of the nanofiber structures and determination of their respective dimensions were carried out by AFM. For this purpose, the supernatant of the suspension was subjected to ultrasound for five minutes. Then, an aliquot of the new supernatant was diluted (1:4), and 10 μL was deposited on mica fixed on a metal disc and air-dried at room temperature. The analysis was conducted on a Cypher ES Microscope (Asylum Research, Santa Barbara, CA, USA), with controlled humidity (40–45%) and temperature (22 °C). A silicon AC240TS tip was used for the scans at a frequency of 70 Hz, and the force constant was set to 2 N min^−1^. Scans were performed in different areas ranging from 10 × 10 μm to 1 × 1 μm at a speed of 1.5 Hz (lines s^−1^). The scanning mode was intermittent contact (Tapping Mode). The acquired images were processed using the manufacturer’s software, AR, version 16.

### 2.5. Characterization of Films

#### 2.5.1. Grammage, Thickness, Moisture Content, and Water Solubility

The grammage of the prepared films was calculated as follows [20]: the film samples were cut (10 × 10 cm) and weighed on an analytical balance. The result was calculated according to the following equation:

(1) G=M/A

where G is the grammage ($g/m^{2}$), M is the mass (g), and A is the area of the film ($m^{2}$).

Film thickness was measured using a micrometer (Mitutoyo, Kawasaki, Japan). Ten measurements were taken at random points in each film. The moisture content was determined using a gravimetric method (drying at 105 °C until constant weight). The water solubility analysis samples were carried out following the method described by Antoniou et al. [21]. Samples were placed in a 250 mL Erlenmeyer with 50 mL of distilled water and shaken at 70 rpm in an orbital shaker at 20 °C for 24 h, followed by filtration and drying of the samples at 105 °C until constant weight. The water solubility was calculated using

(2) $$Solubility(\%)=\frac{W_{0}-W_{1}}{W_{0}}\times 100$$

where $W_{0}$ is the initial dry weight of the sample (g), and $W_{1}$ is the final dry weight of the sample (g).

#### 2.5.2. Water Vapor Permeability Analysis (WVP)

Water vapor permeability was determined gravimetrically according to ASTM standard method E96 [22]. The system contained the sample and a circular capsule with granulated calcium chloride (previously dried in a forced-air oven). The film and the capsule were sealed using beeswax. Then, the system was weighted and rested in a desiccator containing a saturated calcium chloride solution at 21 °C and a relative humidity of 75%. The system was weighed on an analytical scale every 30 min for five hours. The analysis was evaluated in quadruplicate with two repetitions for each film sample. The water vapor permeability (WVP), expressed in g/m s Pa, was calculated using

(3) $$WVP=\left(\frac{W\;}{t}\right)\left(\frac{l}{A∆P}\right)$$

where W/t represents the linear regression calculation of experimental points of weight gain (g) of the capsules as a function of time, l is the thickness of the film, A is the film-exposed area, ∆P is the pressure difference of water vapor to the pure water at 25 °C.

#### 2.5.3. Color Measurement and Opacity

Color analysis was carried out using a Colorflex Hunterlab meter according to the CIELab system. Color parameters a* (red-green), b* (blue-yellow), and L (lightness) of the CIELab scale were determined and further used to calculate chroma (C*) and hue value (h*). Film opacity was determined following the procedure detailed in Almeida et al. [23] and calculated based on the light absorbed by the biocomposites vbionanocomposites (1 mm) at 600 nm, using a UV Spectrophotometer (UV-Micronal, AJX 1900, Micronal S.A., São Paulo, Brazil). Opacity was determined as the ratio of absorbance to film thickness (mm) (Abs600 nm mm^−1^).

#### 2.5.4. Mechanical Properties

The mechanical properties tensile strength (TS) and elongation at break (EB) were measured in a texture analyzer (TAXTPLUS (Stable Micro Systems), according to the ASTM standard method D882-18 [24]. The samples were cut into strips (100 × 25 mm). The initial grip separation was 50 mm, and the speed was set at 10 mm s^−1^. Prior to analysis, the film samples were preconditioned at 50% relative humidity at 23 °C for 48 h.

#### 2.5.5. Stability in Acidic and Alkaline Solutions

This analysis assessed the stability and behavior of the developed films in solutions with different pH, evaluating acidic, basic, and neutral conditions. Samples of 16 mm in diameter were immersed in 20 mL of solutions of hydrochloric acid (pH 3), distilled water (pH 7), and sodium hydroxide (pH 12). The samples were kept immersed at 25 °C for 12 days. Changes in the appearance of the samples were recorded with a Motorola camera with 48 megapixels.

#### 2.5.6. Scanning Electron Microscopy (SEM)

The morphology of the films was examined by SEM using a field emission gun (FEG) scanning electron microscope, FEI brand, Quanta 200 FEG model. Samples were affixed to stubs using conductive carbon tape and metalized with carbon. The detection voltage was 20 kV.

#### 2.5.7. Nuclear Magnetic Resonance (NMR)

Characterizing materials in solid-state NMR enables the direct analysis of insoluble macromolecules such as cellulose and polysaccharides present in cell walls. Solid-state NMR spectra were acquired on a Bruker AVANCE III 400 MHz spectrometer, with a resonance frequency of 100.57 MHz for a ^13^C nucleus. Samples were packed into 4 mm zirconia rotors using a 4 mm Magic Angle Spinning (MAS) probe. Cross-polarization with ramping was carried out with a contact time of 2 ms and a recycle time of 3 s for spectrum averaging, followed by the Total Suppression of Spinning Sidebands (TOSS) sequence involving 4 pulses to eliminate sidebands. The spinning speed around the magic angle was set at 5 kHz, collecting 2048 scans [25].

#### 2.5.8. Fourier Transform Infrared (FTIR) of Nanofibers and Films

A Fourier Transform Infrared (FTIR) spectrophotometer (IRAffinity-1, Shimadzu, Japan) with a scanning wave number range of 4000–400 cm^−1^ was used to obtain the FTIR spectra of the nanofibers and produced films.

#### 2.5.9. Thermogravimetric Analysis (TGA) of Both Nanofibers and Films

Thermogravimetric analysis was performed using Shimadzu TGA equipment (TGA-51 Shimadzu, Kyoto, Japan). About 20 mg of the sample was placed in an aluminum pan with the following operating conditions: nitrogen atmosphere with a flow rate of 50 mL min^−1^; heating rate of 10 °C min^−1^; and temperature range from 25 to 600 °C.

#### 2.5.10. Film Biodegradation

Biodegradability assays are essential to assess the potential of the developed material as an alternative to synthetic packaging, which often requires extended periods for degradation. Biodegradability was analyzed in soil prepared according to ASTM G160-03 [26]. The soil was prepared by mixing three components in equal parts: fertile soil with low clay content, manure, and sand. For three months (90 days), the moisture content and pH of the soil were monitored. After this period, we buried samples in it to evaluate their biodegradability. The biodegradability test was carried out according to the methodology described by Batista et al. [6]. Containers with a depth of 12.5 cm were filled with soil up to 4 cm in height. The test specimens (2 × 2 cm) were buried in the soil at approximately 1 to 2 cm in an aerobic condition. The containers were maintained at room temperature, and the soil moisture was regularly replenished by spraying water. The samples were removed and analyzed daily for five days.

## 3. Results

### 3.1. Characterization of Nanofibers

#### 3.1.1. Yield and Reducing Sugars and Lignin Contents

Table 1 shows the results obtained for yield, reducing sugars, and lignin contents. The alkaline treatment was the most efficient treatment for removing non-cellulosic components, accounting for the removal of approximately 65% of these components. This medium promotes solubilization and dissolution, especially of lignin. Still, other components can be solubilized entirely, such as pectin, or partially solubilized, as in the case of hemicellulose since the ester linkages of carboxylic acids are broken [27]. Additionally, the alkaline treatment breaks the hydrogen bonds in the amorphous structure of cellulose. This behavior is essential for increasing the surface area of the crystal and reducing lipid materials such as waxes and oils that cover the fiber [18]. 

Chelating and bleaching treatments removed only 7.5% of the non-cellulosic components in the sample. While chelating treatment aims to remove metal ions, bleaching is applied to complement the delignification stage, turning the color of the sample to lighter hues by removing colored compounds bound to the polysaccharide matrix. Recent studies highlight bleaching as an essential chemical treatment to complement delignification, removing amorphous materials and producing predominantly cellulosic material [28]. After bleaching, the nanofibers were extracted in the enzymatic hydrolysis stage, aiming for better efficiency in disintegrating the sample and obtaining the nanofibers.

After 24 h of enzymatic incubation, the solution containing nanofibers was used to quantify its total reducing sugars content. This study observed a decrease in the reduction in sugar content of approximately 50.6% compared to the untreated original sample. This reduction is mostly due to the elimination of the monosaccharides comprising the hemicellulose fraction.

Pre-treatment with a strong base primarily removes lignin, aiming to obtain a sample rich in cellulose and more prone to fibrillation. Table 1 shows the lignin content in IM (untreated) and the samples after chemical, enzymatic, and mechanical treatments (nanofibers). Approximately 30% of the lignin was removed compared to the initial sample, with only 2% remaining in the nanofibers (after enzymatic treatment). It can be observed that the most significant removal occurred after alkaline treatment and bleaching of the sample, highlighting the efficiency of these protocols in obtaining nanofibers.

It is relevant to emphasize that the alkaline treatment solubilizes lignin and a portion of hemicellulose. In the alkaline treatment, the ester bonds that link lignin to hemicellulose are broken, and the network is disrupted, allowing the solubilization of lignin components. At the same time, alkalis facilitate the breaking of C-H bonds in lignin, generating free radicals, which will subsequently attack the sample matrix, contributing to the further breakdown of the lignin structure. On the other hand, the cellulose fraction usually remains unaffected due to its higher chemical resistance to such treatment [29].

After enzymatic hydrolysis, the nanofiber residue underwent mechanical treatment to comminute the fibers into nanometer sizes. The nanometer size of the fibers produced was corroborated by atomic force microscopy analysis, and the results are presented in Section 3.1.2. Combining enzymatic and mechanical treatment allows nanofibrillation without compromising the material’s thermal stability [30].

#### 3.1.2. Atomic Force Microscopy—AFM

**Figure 1:**
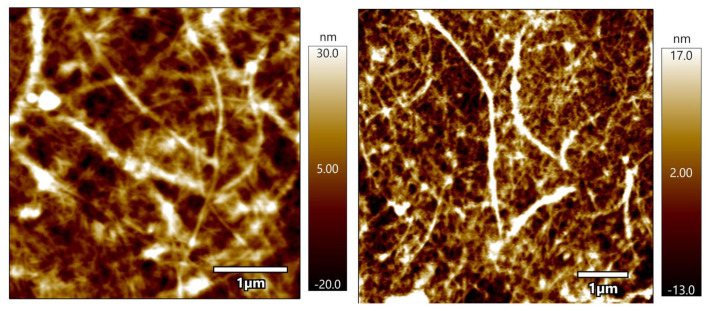
AFM micrographs of obtained cellulose nanofibers.

Figure 1 presents the AFM images obtained for the produced nanofibers. The images reveal a network structure, displaying long entangled cellulose filaments, oriented in various directions. The fibrillar structures had an estimated average length of 118 nm and a diameter ranging from 2 to 30 nm, with an average diameter of 5 nm.

Widiarto et al. [31] prepared cellulose nanofibers from cassava peels by mechanical treatment and observed fiber and needle-like shapes with an approximate size of 100 nm and a diameter ranging between 6.7 and 8.2 nm. Travalini et al. [12] prepared lignocellulose nanofibers from peach palm residues by mechanical defibrillation, with diameters ranging from 4.5 to 12.3 nm, and incorporated the nanofibers produced thereof into cassava peel starch films [4]. It was observed that during the separation of nanofibers in the mechanical process, the suspension behaved like a gel. This can be attributed to the individualization of nanofibers but also suggests the presence of long and entangled nano-sized cellulose fibers [5], characteristics observed in the present study.

Fibers at nanoscales are recommended to enhance the strength of polymeric materials. However, it is essential to consider that the reinforcing ability of nanofibers is also associated with other factors, such as the compatibility between fibers and matrix components and the concentration of nanofibers [16].

A high aspect ratio is desirable for better efficiency as a reinforcing material because, in these conditions, there will be a favoring of energy transfer through the nanofiber–matrix interface. In this study, an aspect ratio (length/diameter) of approximately 23.6 was observed, which is lower than that reported by Leite and co-workers [17], who found an aspect ratio of 58.8 to 77.3 in nanofibers obtained from cassava peelings and roots. Differences in the nanofiber extraction procedures can explain this difference. The higher values observed in that study [17] can be ascribed to the use of acid hydrolysis for nanofiber breakdown, as opposed to the use of enzymes for hydrolysis in the present study. The experimental protocol is one factor affecting the nanofiber’s aspect ratios. Additionally, cassava periderm and cortex were used in this study, which could be another factor associated with the observed differences.

#### 3.1.3. Fourier Transform Infrared (FTIR)

**Figure 2:**
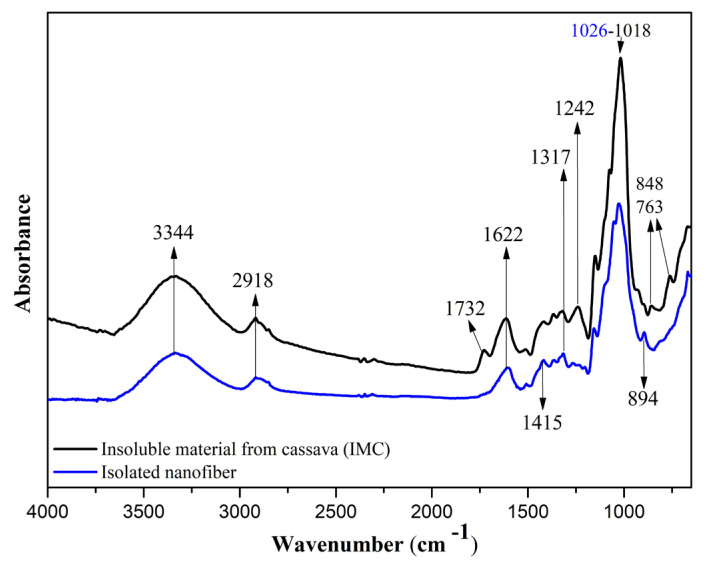
Spectra of FTIR for insoluble material from cassava periderm and cortex (IM) and isolated nanofibers.

Figure 2 shows the spectra obtained for the IM and nanofiber samples. IM (black spectrum) exhibited a broader band in the region of 3500 to 3200 cm^−1^, indicating the O–H stretching vibration of the polysaccharides’ molecules. There was a reduction in the intensity of this band in the nanofiber sample (blue spectrum). This change in intensity occurred due to the removal of the hemicellulose fraction of the cassava periderm and cortex. In addition, this band and that observed at 1622 cm^−1^ in both samples can be attributed to the presence of water [17,32].

The band at 2918 cm^−1^ is related to C–H stretching, more intense in the IM sample spectrum, due to a higher amount of the amorphous fraction of cellulose. Thus, it can be inferred that the pretreatment and enzymatic hydrolysis reduced the amorphous fraction in the nanofiber sample [32]. The band observed at 1732 cm^−1^ for the IM sample corresponds to the C=O group, typical of lignin and hemicellulose. This band does not appear in the nanofiber sample, indicating that pre-treatment and enzymatic hydrolysis effectively remove these constituents. These findings are consistent with similar spectra observed in the literature [32,33].

The IM sample exhibited a broad band at approximately 1427 cm^−1^; however, the sample after the applied treatments showed a shifted band at 1415 cm^−1^. This shift corresponds to the vibration of CH_2_ bonds attributed to crystalline cellulose. These intermolecular bonds occur at the C6 position of the molecule [34,35].

The band at 1318 cm^−1^ was attributed to C–H vibrations in cellulose, lignin, and hemicellulose, following its higher intensity in IM [16,17,34]. The band at 1242 cm^−1^ was observed only in IM and was attributed to the presence of phenolic groups and derivatives, characteristic of lignin [17,32,33]. The band observed at 894 cm^−1^ is attributed to β-glycosidic linkages between glucose units in cellulose [31,34].

The results observed in the FTIR spectra demonstrate that the chemical and enzymatic pre-treatment, combined with physical treatment, enabled the isolation of nanofibers from the insoluble material obtained from cassava periderm and cortex polysaccharide extraction, with the efficient removal of lignin and hemicellulose, with a corresponding increase in the amount of cellulose in nanofibers.

#### 3.1.4. Thermal Analysis

**Figure 3:**
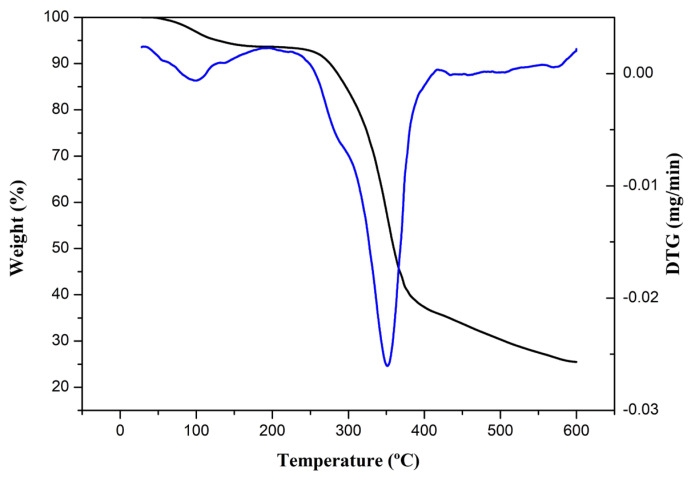
Thermogram of isolated nanofibers: TGA (black) and first derivatives (blue).

The thermograms of the isolated cellulose nanofibers are presented in Figure 3, and two stages of thermal degradation are observed. The initial stage is associated with the presence of moisture in the nanofibers [28]. In contrast, the second stage can be related to the decomposition of hemicellulose and cellulose since the event began at 245 °C and ended at 386 °C. The final degradation temperature suggests a higher percentage of crystalline regions in the obtained nanofibers. Celluloses with a predominance of crystalline regions tend to degrade at maximum temperatures around 380 °C [36].

From the second stage onwards, no other events were observed. This aligns with the data observed in the FTIR analysis, indicating lignin removal in the nanofibers since lignin has a higher decomposition temperature than cellulose. Lignin initially degrades at approximately 380 °C and can complete its degradation up to 500–600 °C [31].

### 3.2. Characterization of Films

#### 3.2.1. Grammage, Thickness, Moisture Content, and Solubility

The data presented in Table 2 are essential for understanding potential changes when adding nanofibers to the formulation. Grammage values are related to weight per unit area and significantly impact mechanical properties; a higher basis weight often results in a stronger material [37].

The results demonstrate no significant differences in the grammage of the films, with a slight decrease as the nanofiber concentration increased. The justification for this behavior might be related to the size of the nanofibers. Although microscopic analysis revealed a nanoscale size for the nanofibers, the variations in diameters and lengths could have led to larger particles that are more difficult to evenly distribute within the matrix [38]. Furthermore, they may have resulted in the formation of void spaces in the material [37], which led to a decrease in the density of the films (BC) from 2.53 g/cm^3^ to 1.62 g/cm^3^ in the formulation with the highest nanofiber concentration (BN2.5). Based on this behavior, it is possible to understand why the film thickness increased with the increase in nanofiber concentration. The control film probably had fewer void spaces, leading to higher density and reduced thickness. 

For the moisture analysis, Table 2 indicates that incorporating nanofibers increased the control formulation’s moisture content. However, it is noticeable that this increase was not proportional to the nanofiber concentration, as there was a parabolic trend with a maximum point followed by a reduction in moisture content with increasing nanofiber content. This result is consistent with the thermal analysis conducted in this study, which exhibited a similar behavior. A low concentration of nanofibers was possibly insufficient to facilitate a higher number of intermolecular bonds, thereby maintaining the exposure of hydroxyl groups in starch, which is hydrophilic. As the nanofiber concentration increased, more intermolecular bonds formed among nanofibers, starch, galactomannans, and glycerol. Consequently, there was a reduction in the total available hydroxyl groups for interaction with water [35]. Furthermore, the nanofibers remained partially clustered, as observed in the microscopy analysis. Hydrogen bond interactions amongst nanofibers were favored during film preparation instead of interactions of nanofibers with water molecules [4].

Regarding the solubility analysis, it is observed that the incorporation of nanofibers and the increase in their concentration in the formulations led to a significant reduction in this parameter. The same behavior was reported in previous studies [4,39] in association with the preference of nanofibers to form bonds with other matrix constituents instead of breaking them to bind with water. This behavior can also be attributed to the creation of a network of nanofibers, hindering the diffusion of water into the polymeric matrix.

#### 3.2.2. Color Measurement and Opacity

Table 3 demonstrates a notable decrease in L * values with nanofibers’ addition and incremental concentration. These outcomes are consistent with findings by Lago et al. [9,40] and Martins et al. [4], who incorporated nanofibers into cassava-starch-based films, observing a decrease in luminosity values as the nanofiber concentration increased due to their light-blocking properties.

Concomitant with the addition and increased concentration of nanofibers, the chroma * and hue * parameters exhibited an elevation, signifying an enhanced saturation and yellow hue intensity in the materials.

Similarly, opacity demonstrated a proportional increase with rising nanofiber concentration. These findings align with the results reported by Silveira et al. [41], who developed cellulose-nanofiber-based active films with cassava starch and Melaleuca essential oil, recording opacity values ranging from 0.33 to 0.50.

It is important to consider that certain food products should avoid exposure to light due to their high lipid content, making them susceptible to rancidity and other oxidation reactions. In such cases, minimizing light transmission becomes crucial. Nanofibers protect packaging materials by establishing robust interactions with cellulose, enhancing opacity and light dispersion [42]. The observed behavior in opacity analysis aligns with the decreased luminosity values, indicating reduced transparency in the examined samples. However, this increased opacity does not compromise visibility and holds potential for diverse applications without adverse effects.

#### 3.2.3. Mechanical Properties and Water Vapor Permeability (WVP)

Incorporating nanofibers in the film led to a slight reduction in the tensile strength of the bionanocomposites (Table 4). However, this effect was not significant (*p* < 0.05) up to a nanofiber concentration of 1.5% (Table 4). No statistical difference was observed in the elongation percentage, even with the addition of nanofibers in the studied formulations with values in the 41 to 45% range. 

These results demonstrate that the bionanocomposites, developed with starch and nanofibers extracted from cassava peel and pulp, galactomannans, and glycerol, exhibited good tensile strength while maintaining material flexibility. Literature reports indicate that increasing the concentration of nanofibers can improve tensile mechanical properties up to a specific concentration. The enhanced reinforcement is primarily due to the stronger bonds that cellulose forms with matrix constituents, creating a rigid network. However, beyond a certain threshold, agglomeration may occur, leading to a significant decrease in tensile strength [43].

Improvements in film mechanical properties due to the inclusion of cellulose nanofibers are achieved when there is a strong physicochemical interaction between the added nanofibers and the film matrix, with the interacting nanofibers facilitating stress transfer in the matrix. This was not the case in our study. The herein moderate interactions between the nanofibers and the polysaccharide matrix might be related to the fact that both the starch and the galactomannan employed were highly branched, with 83% amylopectin in starch [15] and a mannose-to-galactose ratio of about 4:1 in galactomannan. Both polysaccharides are moderately soluble in water at room temperature. Since cellulose is not soluble in water, interactions between starch and galactomannan are more easily accomplished than their interactions with crystalline cellulose nanofibers. Cassava starch in general, be it from the peel or from the bagasse, was identified as an A-type allomorph [44,45], with a crystallinity index of about 35%. As the starch is solubilized together with locust bean galactomannan, recrystallization to its original morphology does not occur, due to interactions between the two distinct polysaccharides. Therefore, the packing density of the film prepared thereof will be lower than the packing densities of the original polysaccharides, allowing for the cellulose nanofibers to occupy intermolecular spaces and structural voids in a way that will not contribute to an enhanced stress transfer but can contribute to an enhanced water vapor barrier. Although the mechanical properties were not enhanced with the incorporation of cellulose nanofibers, the films still ended up being stronger than some films produced from petroleum-derived products, such as polyethylene, which presents an average tensile strength of 4.6 MPa [46]. This result is considered satisfactory and demonstrates the potential of the materials produced in this study as substitutes for non-renewable source materials.

Regarding WVP, it was observed that the bionanocomposites were significantly less permeable to vapor than the biocomposite, and WVP decreased as the concentration of nanofibers increased. This behavior strongly suggests that the incorporation of nanofibers improved the film’s barrier properties, which is significant as the goal is to decrease the exchange of water between the product and the environment. Hence, the lower the permeability of the packaging material, the longer the product’s durability, given that moisture content is a decisive factor for microbial growth. This reduction in WVP is attributed to a polymer network with a certain degree of intermolecular interactions and a high degree of molecular orientation, which likely reduces the diffusion coefficient by restricting chain mobility, creating entanglement and blockages that hinder the passage of water molecules. This is also consistent with the microscopic analysis of the nanofibers and with previous studies [47].

Materials with low water vapor permeability values are essential for the packaging of dry products, where contact with moisture must be restricted, and for coating high-respiration-rate vegetables, which degrade due to water loss [9]. The bionanocomposites produced in this study can be considered as having a moderate barrier, as their values fall within the range of 0.004 to 0.4 g mm/m^2^ h kPa established in the literature [48], so they could be directed towards packaging applications for moderately moist foods [46]. Another possibility would be blending with paper, thereby further reducing water permeation and preventing product deterioration. Furthermore, it is important to note that the results from this study are not limiting and can be considered promising. This is especially true considering the diversity in food products with varying gas exchange requirements.

#### 3.2.4. Stability in Acidic and Alkaline Solutions

The stability of the prepared films under acidic and alkaline pH conditions was evaluated, considering that this is an essential parameter for defining their applications (Table 5). The control formulation swelled approximately 1.5 times, while the sample with a 0.5% addition of nanofibers showed a swelling of 1.4 times under acidic conditions. The samples incorporated with 1.5 and 2.5% nanofibers swelled about 1.3 times. The results suggest that adding nanofibers improved sample stability under acidic conditions. 

Furthermore, after 12 days of immersion, the samples remained intact without visually noticeable cracks. These results indicate the possibility of directing the bionanocomposites for application in acidic food packaging, such as processed meats and some fruits. The acidic environment did not weaken or break the intermolecular and intramolecular bonds formed between the constituents of the polymer matrix in these formulations during the evaluated period.

In the alkaline medium, the control sample swelled 1.5 times, and the samples incorporated with nanofibers, regardless of the concentration, showed a 1.3-fold increase in diameter compared to the initial diameter (16 mm). Based on the previous data, adding nanofibers probably allowed moderate interactions between nanofibers and the other constituents of the polymer matrix. As a result, sodium hydroxide was not strong enough to break these bonds and react with the hydroxyl groups of starch. This behavior was not observed in the formulation without the addition of nanofibers. Therefore, it is possible that the starch chains were more exposed to disintegration in this medium, as sodium hydroxide reacted more easily with the hydroxyl groups of the starch molecule, breaking the hydrogen bonds that would form inter- and intramolecular interactions. It is important to note that all the evaluated samples exhibited greater stability in the studied media than other starch films reported in the literature [49].

#### 3.2.5. Scanning Electron Microscopy (SEM)

**Figure 4:**
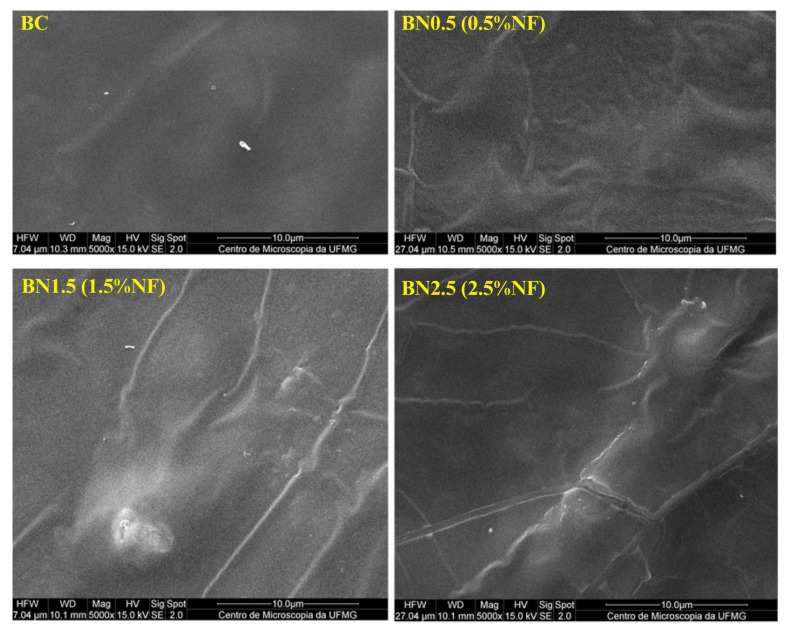
SEM micrographs (5000×) of surfaces of the BC and bionanocomposites BN0.5, BN1.5, and BN2.5, containing 0.5, 1.5, and 2.5% of nanofibers, respectively.

The surface morphology of the biocomposite and bionanocomposites was examined using scanning electron microscopy, and the images obtained are presented in Figure 4.

The formulation without the addition of nanofibers presented a homogeneous surface. However, upon incorporating 0.5% of nanofibers, the surface became rough. One explanation for the observed effect is that the quantity of 0.5% nanofibers was insufficient to establish hydrogen bonds, so the fibers remained between the polymer chains. This observation is in line with the data from the thermogravimetric analysis. The behavior changes as the content of nanofibers increases. It was observed that the matrix provides better coverage for the nanofibers, reducing the surface roughness [12]. Presumably, the nanofibers were well dispersed in the matrix, owing to the increase in hydrogen bonds [35]. Such bonds can reduce accessible OH groups, providing this coverage; this effect might improve water vapor permeability. This result agrees with the data obtained from the moisture and WVP analyses. On the other hand, isolated agglomerated nanofibers were observed at the maximum studied nanofiber content (2.5%). A higher concentration may render the material less cohesive [50]. This explains the observed behavior regarding the mechanical properties. The nanofibers form stronger bonds with the matrix constituents up to a certain concentration, creating a rigid network. However, with a 2.5% addition in nanofiber content (regarding the control film), agglomeration of the nanofibers was favored, and it was precisely this agglomeration that reduced the strength of materials with added nanofibers [43].

#### 3.2.6. Fourier Transform Infrared (FTIR)

**Figure 5:**
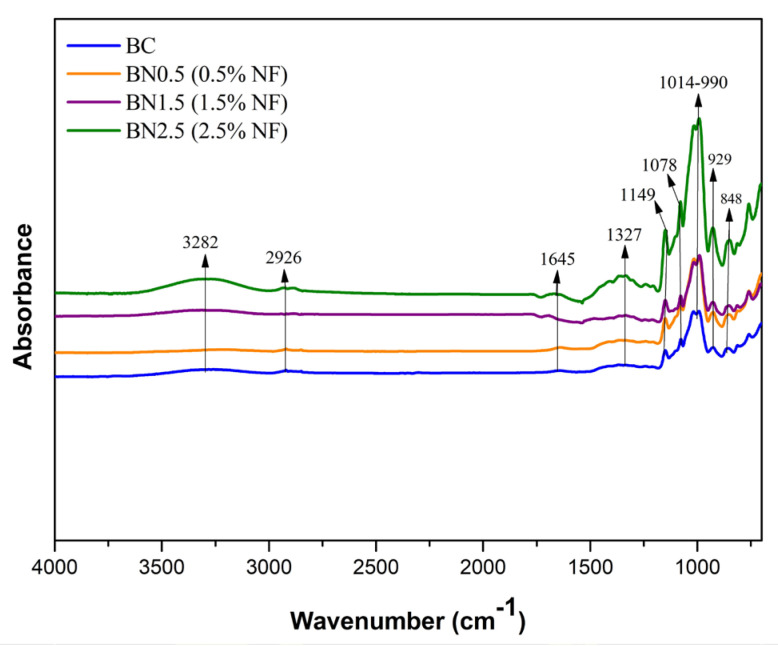
FTIR spectra of the BC and bionanocomposites BN0.5, BN1.5, and BN2.5, containing 0.5, 1.5, and 2.5% of nanofibers, respectively.

Figure 5 shows the FTIR spectra obtained for the prepared films. The bionanocomposites did not show any shift or presented new bands compared to the biocomposite developed solely with starch and galactomannan. All formulations exhibited spectra similar to those reported for starch, galactomannans, and cellulose structures.

The band between 3000 and 3700 cm^−1^ corresponds to the vibration of the –OH group predominant in the polysaccharides’ structures and the water–water and water–biopolymers interactions [51]. Additionally, the band at 3282 cm^−1^ can be attributed to –OH stretching in cellulose. It is observed that the intensity of this vibration is higher at the maximum concentration of added nanofibers (green curve), indicating more hydrogen bonds between the compounds and a good interaction of the nanofibers in the matrix, associated with an increase in the cellulose content. This increase shows the efficiency of the applied pretreatments for nanofiber extraction [12].

Figure 5 shows a slight vibration around 2926 cm^−1^, attributed to –CH stretch vibrations in the central molecule of cellulose [52]. A band with lower intensity was observed at 1645 cm^−1^, attributed to water absorbed by the polymers. Bands in the range of 1654–1662 cm^−1^ have been mentioned in previous studies in association with water absorption by starch molecules [4,9,12]. 

The spectral region typical of polysaccharides ranges from 1300 to 900 cm^−1^ and is known as the fingerprint region [53]. Although there was no structural alteration in this region with the addition of nanofibers, there was an increase in band intensities. A band at approximately 1078 cm^−1^ was observed in all samples, which can be attributed to the stretching of C–OH bonds. Lago et al. [9] reported a band at 1077 cm^−1^ related to the stretching of the C–OH bond in starch and galactomannan molecules, as well as the C–O stretching in cellulose, hemicellulose, and lignin, thus explaining the increase in intensity with the addition of nanofibers.

The bands observed at 1014, 990, and 926 cm^−1^ are attributed to the stretching of C–O bonds in the anhydroglucose ring of starch; C–O stretching in cellulose, hemicellulose, and lignin; and vibrational modes related to the CH_2_ group, respectively, and they are characteristic of starch films [9,12]. The bands at approximately 848 and 806 cm^−1^ with lower intensity are attributed to the α-D-galactopyranose and β-D-mannopyranose units, respectively [39]. These constituents are part of the galactomannan chain.

#### 3.2.7. Thermal Analysis

**Figure 6:**
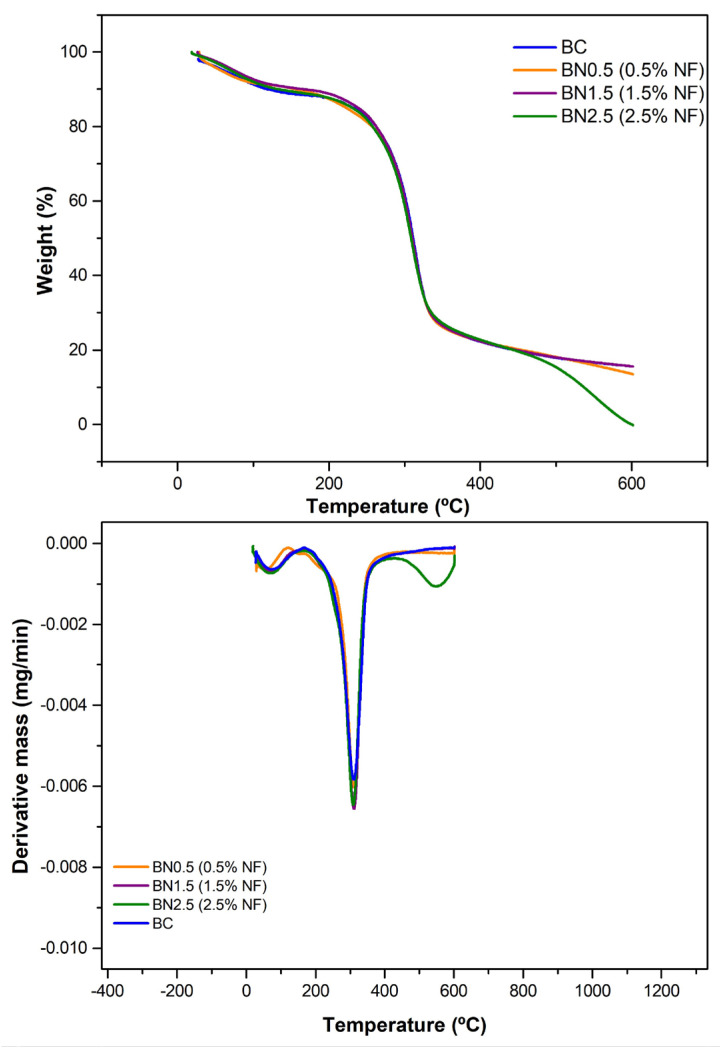
Thermogravimetric (TGA) and derivative thermogravimetric (DTG) curves of the BC and bionanocomposites BN0.5, BN1.5, and BN2.5, containing 0.5, 1.5, and 2.5% of nanofibers, respectively.

The biocomposite and bionanocomposites were analyzed thermogravimetrically and the thermograms are presented in Figure 6. This analytical determination is essential in characterizing biodegradable materials, such as those studied in this research, as it assesses stability when subjected to temperature variations. The addition of 2.5% nanofibers in the formulation was the only one that presented a difference compared to the other formulations, which maintained a relatively unaltered thermal behavior as observed from the DTG curves (Figure 6).

All samples exhibited mass loss at temperatures below 100 °C, corresponding to the elimination of water and other eventual volatile components. Notably, part of this water is trapped in starch molecules due to their hydrophilicity, caused by hydrogen bonds formed between hydroxyl groups and glucose units. This can also occur with galactomannans and empty spaces within the polymeric chain. The presence of water absorbed by the films is consistent with the FTIR spectra, where the band in the region of 1645 cm^−1^ was observed (Figure 5).

The incorporation of 0.5% nanofibers into the film matrix decreased the initial degradation temperature (119.34 °C) of the second event; however, this behavior was not maintained. As more nanofibers were incorporated into the formulation, the degradation temperature increased again; 1.5 and 2.5% nanofibers exhibited degradation temperatures of 144.79 °C and 157.67 °C, respectively. At low concentrations, the nanofibers may allocate in-between polymeric chains, reducing the flexibility of the amylopectin chains and preventing a stronger interaction among all sample constituents (water–starch–glycerol–galactomannan–nanofibers). This behavior is consistent with the mass loss of this sample, which was higher at this stage [54].

A single thermal degradation stage was observed except for the sample with a 2.5% nanofiber addition and excluding the initial stage corresponding to the removal of water from the samples. This indicates that chemical bond breakage occurred simultaneously, suggesting similar interactions during film formation. Other studies in the literature have reported this behavior [41,55].

On the other hand, the thermal degradation of the bionanocomposite with 2.5% nanofibers in the formulation showed an additional stage. The increase in nanofiber concentration might have favored stronger chemical bonds, requiring higher energy for degradation. For the third degradation stage, an initial temperature of 412.35 °C was observed with a mass loss of 21.72%, producing less than 1% of inorganic material. This mass loss could correspond to the removal of more thermally stable compounds, the depolymerization of high-molecular-weight constituents, and the rupture of intense chemical bonds formed during film preparation [56]. The same behavior has been observed in other studies aimed at characterizing materials for packaging with the addition of nanofibers. In these studies, the increase in nanofiber concentration led to additional thermal degradation stages [57,58].

#### 3.2.8. Nuclear Magnetic Resonance (NMR)

**Figure 7:**
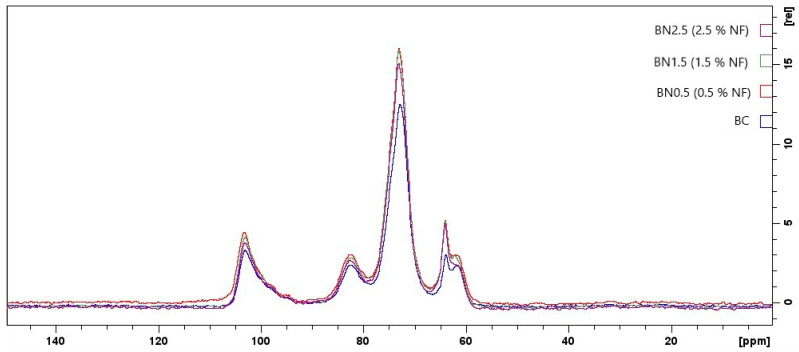
^13^C NMR spectra obtained for the biocomposite, BC, and bionanocomposites BN0.5, BN1.5, and BN2.5.

Figure 7 shows the ^13^C NMR spectra for the biocomposite and bionanocomposites produced in this study. Although no new signals have emerged, the ^13^C NMR spectra for the bionanocomposites revealed slight changes in chemical shifts compared to the biocomposite spectrum. This observation indicates that the nanofibers likely interacted with the other constituents of the polymeric matrix [59].

Starch gelatinization occurs during the formation of the biocomposite and bionanocomposites, in which its ordered structure is lost, and native starch is converted into a thermoplastic polymer. It can undergo rearrangement either in the starch chain or the chain formed by interacting with other constituents in the polymeric matrix. This rearrangement is called polymorphism, either Type A (the crystals form a monocyclic network) or Type B (the crystals form a hexagonal network). They are usually associated with triplets or doublets in the C1 resonance in the spectra [60]. However, in this study, we did not observe triplets or doublets resonating at C1, suggesting the formation of a disordered phase in this region. This is possibly due to the lower mobility of starch chains. Hydrogen bonds are not formed between starch chains but among the hydroxyl groups of starch and polar groups in the plasticizer, galactomannan, and/or cellulose. This behavior corroborates the data from the present study’s water vapor permeability and thermogravimetric analysis. In these analyses, a higher concentration of nanofibers promotes stronger interactions, and the glycerol remains accumulated on the surface of nanofibers, contributing to the crystallinity of amylopectin. Thus, a crystalline network is formed around it, restricting its mobility. The signals attributed to C1 correspond to the anomeric carbon for α-D-glucose and are associated with the structure of amylose [59].

A signal at approximately 82 ppm can be observed for C4, which is typical of the crystalline region. Zheng et al. [61] also observed a signal in the range of 80–84 ppm, and they attributed it to an ordered structure formed by type V complexes, which reduces the degree of helicity of amylose. This is consistent with the obtained C1 signal.

C1 is on the side corresponding to the lower energy field, while C6 is on the higher-energy-field side [62]. C2, C3, and C5 fall between these extremes, and their signals were associated with spectra at approximately 72 ppm and attributed to the HC–O groups [63]. Smits et al. [64] observed the interaction between starch and plasticizer in the ^13^C NMR spectra. They detected a signal around 72 ppm, which was attributed to the central carbon of glycerol. The authors stated that these signals can overlap.

Carbon C6 appears as a doublet, attributed to the CH_2_–OH groups of cellulose at 63 ppm. It corresponds to type II cellulose in the range between 61.72 and 62.03 ppm. When nanofibers are added, and their concentration is increased in the formulations, it is observed that the signal at 63.75 ppm shifts to the lower field. The lower field order follows the number of electronegative atoms. In this way, oxygen atoms from the free anomeric carbon in starch may provide higher electronegativity to the control sample. On the other hand, when the oxygen atoms from the anomeric carbon participated in intramolecular hydrogen bonds upon incorporating nanofibers into the films, this electronegativity was reduced [64,65].

The signal around 62 ppm falls within the range of 58–66 ppm, which has been observed in some studies with cellulose. These studies reported that this range is associated with C6 [66]. Additionally, the doublet at C6 exhibits a higher-intensity signal associated with the crystalline region and a shoulder corresponding to the amorphous region [67]. Analyzing the data, it was possible to observe that when increasing the nanofiber concentration to 2.5%, the shoulder on this carbon disappears, indicating a greater crystalline zone for this sample. This result is consistent with the data from thermal analysis, where a higher energy content was required to break the crystalline structure of the sample.

#### 3.2.9. Film Biodegradability

**Figure 8:**
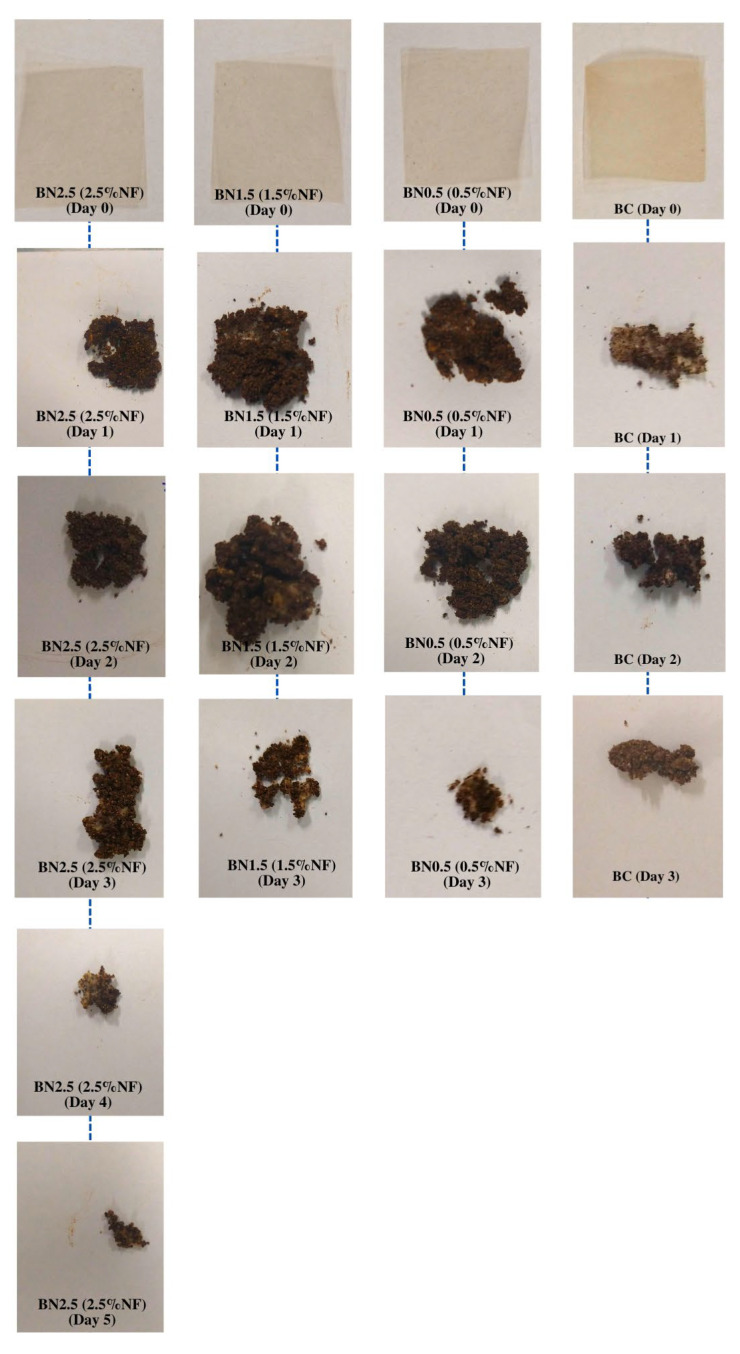
Biodegradability results for the biocomposite, BC, and bionanocomposites BN0.5, BN1.5, and BN2.5.

It is possible to observe that both the formulation without nanofibers (biocomposite) and the bionanocomposites incorporated with 0.5 and 1.5% of nanofibers degraded entirely on the fourth day of evaluation (Figure 8). As the concentration of nanofibers was increased to 2.5%, there was a need for two more days for complete degradation of the sample. This behavior is possibly due to the crystalline structure of cellulose and its higher quantity in the film, which formed a barrier against water molecules, preventing the propagation of cellulose-degrading enzymes and other nearby constituents [68].

These findings are consistent with other studies in the literature that utilized cassava in the composition of packaging materials [5,47,68]. In summary, the prepared biocomposite and bionanocomposites were deemed biodegradable and promising alternatives for minimizing environmental impacts caused by the disposal of synthetic materials. They can also potentially reduce public spending required to treat these generated wastes.

## 4. Conclusions

In this study, nanofibers were prepared from cassava waste. Their potential for application in packaging materials was explored due to their good thermal and chemical stability. Based on this, bionanocomposites were formulated, and their performance was compared to that of a control formulation (without incorporating nanofibers). The results showed that adding the reinforcing material provided greater thermal stability and improved water vapor permeability. Moreover, the bionanocomposites remained stable in acidic and alkaline pH conditions during a 12-day test period. All studied formulations are biodegradable within seven days, contributing to the reduction in environmental impacts caused by the improper disposal of cassava waste and synthetic packaging. Therefore, this study offers a biomaterial with potential eco-friendly applications. This research represents an essential strategy in developing a material with potential applications in the food industry and beyond. Furthermore, we recommend that future research explores the use of more readily available enzymes, allowing for the production of reinforcement materials through a simpler manufacturing process with reduced costs, facilitating their industrial production.

## Figures and Tables

**Table 1:** Results obtained for cellulose nanofiber yield after chemical and enzymatic pre-treatments and reducing sugars and lignin contents.

Parameter and Treatment	Result
Yield after alkaline treatment (%)	34.6
Yield after bleaching (%)	92.5
Yield after enzymatic hydrolysis (%)	46.0
Reducing sugar (mg/mL)	2.53 ± 0.01
IM (% lignin)	30.10 ± 1.95 ^a^
Bleaching sample (% lignin)	4.32 ± 1.17 ^b^
Nanofibers (% lignin)	2.10 ± 0.28 ^b^

Yield data initial sample mass of 50 g. The remaining results are expressed as mean ± standard deviation (*n* = 3). Different letters in the same column represent statistical differences in the results according to Tukey’s test (*p* < 0.05).

**Table 2:** Values obtained for grammage, thickness, moisture content, and solubility for the films that were produced.

Sample	Grammage g/m^2^	Thickness (mm)	Moisture (%)	Solubility (%)
Biocomposite				
BC	50.34 ± 2.67 ^a^	0.079 ± 0.010 ^b^	10.99 ± 0.10 ^b^	70.35 ± 0.02 ^a^
Bionanocomposite				
BN0.5 (0.5% NF)	48.14 ± 10.93 ^a^	0.093 ± 0.010 ^b^	13.00 ± 0.06 ^a^	18.85 ± 1.12 ^b^
BN1.5 (1.5% NF)	40.70 ± 6.43 ^a^	0.096 ± 0.012 ^ab^	12.37 ± 0.44 ^a^	15.28 ± 0.85 ^c^
BN2.5 (2.5% NF)	38.93 ± 7.54 ^a^	0.097 ± 0.010 ^a^	12.39 ± 0.35 ^a^	15.14 ± 0.90 ^c^

Results are expressed as mean ± standard deviation (*n* = 3). Different letters in the same column indicate statistically significant differences using Tukey’s test (*p* < 0.05).

**Table 3:** Optical properties (color and opacity).

Sample	L	C *	h *	Opacity
Biocomposite				
BC	88.35 ± 0.005 ^a^	7.67 ± 1.32 ^a^	−88.47 ± 1.23 ^a^	0.37 ± 0.05 ^a^
Bionanocomposite				
BN0.5 (0.5% NF)	84.04 ± 0.015 ^b^	15.06 ± 0.040 ^b^	85.19 ± 0.02 ^b^	0.40 ± 0.01 ^a^
BN1.5 (1.5% NF)	81.65 ± 0.020 ^c^	16.31 ± 0.26 ^c^	85.26 ± 0.01 ^c^	0.41 ± 0.01 ^a^
BN2.5 (2.5% NF)	79.07 ± 0.010 ^d^	19.71 ± 1.56 ^d^	85.37 ± 0.14 ^d^	0.66 ± 0.02 ^b^

* Results are expressed as mean ± standard deviation (*n* = 3). Different letters in the same column indicate statistically significant differences using Tukey’s test (*p* < 0.05).

**Table 4:** Mean values and standard deviations of tensile strength (TS), elongation at break (EB), and water vapor permeability (WVP).

Sample	TS (MPa)	EB (%)	WVP (g mm/m^2^ h kPa)
Biocomposite			
BC	10.38 ± 0.72 ^a^	41.10 ± 3.54 ^a^	0.15 ± 0.01 ^a^
Bionanocomposite			
BN0.5 (0.5% NF)	10.30 ± 3.08 ^a^	45.1 ± 12.2 ^a^	0.11 ± 0.02 ^b^
BN1.5 (1.5% NF)	9.52 ± 1.81 ^ab^	45.3 ± 3.8 ^a^	0.060 ± 0.003 ^c^
BN2.5 (2.5% NF)	6.57 ± 1.04 ^b^	45.3 ± 1.7 ^a^	0.055 ± 0.007 ^c^

Results are expressed as mean ± standard deviation (*n* = 3). Different letters in the same column indicate statistically significant differences using Tukey’s test (*p* < 0.05).

**Table 5:** Mean values and standard deviations of sample diameter (*n* = 3) in acidic and alkaline solutions (original diameter = 16 mm).

	Diameter (mm)	
Sample	pH 3.0	pH 10
Biocomposite		
BC	23.5 ± 0.5	23.2 ± 1.6
Bionanocomposite		
BN0.5 (0.5% NF)	21.4 ± 1.1	20.3 ± 0.5
BN1.5 (1.5% NF)	21.0 ± 0.5	20.0 ± 0.3
BN2.5 (2.5% NF)	20.7 ± 0.9	19.9 ± 0.2

## Data Availability

The data presented in this study are available upon request from the corresponding author.
